# Impact of Electronic Health Record Interoperability on Telehealth Service Outcomes

**DOI:** 10.2196/31837

**Published:** 2022-01-11

**Authors:** Xinyue Zhang, Richard Saltman

**Affiliations:** 1 Department of Health Policy and Management Rollins School of Public Health Emory University Atlanta, GA United States

**Keywords:** Electronic Health Records, Telehealth, Telemental health, Pandemic, Health outcomes, Health Policy

## Abstract

This paper aims to develop a telehealth success model and discusses three critical components: (1) health information quality, (2) electronic health record system quality, and (3) telehealth service quality to ensure effective telehealth service delivery, reduce professional burnout, and enhance access to care. The paper applied a policy analysis method and discussed telehealth applications in rural health, mental health, and veterans health services. The results pointed out the fact that, although telehealth paired with semantic/organizational interoperability facilitates value-based and team-based care, challenges remain to enhance user (both patients and clinicians) experience and satisfaction. The conclusion indicates that approaches at systemic and physician levels are needed to reduce disparities in health technology adoption and improve access to telehealth care.

## Executive Summary

A telehealth platform integrated with an interoperable electronic health record (EHR) system can contribute directly toward achieving the often-discussed “quadruple aim” [1]—better health outcomes, improved patient experience, lower costs, and improved clinician experience. This paper develops a telehealth success model and discusses three critical components: (1) health information quality, (2) EHR system quality, and (3) telehealth service quality to ensure effective health care service delivery, reduce professional burnout, and enhance access to care.

Despite the benefits of telehealth in rural health, mental health, and Veterans Administration health services, disparities continue to exist in access to care. Patients without internet service, appropriate devices, or digital literacy skills experience greater challenges in accessing care via telehealth. The COVID-19 pandemic has also caused substantial financial strain on hospitals, and it is uncertain if the current reimbursement and payment model for telehealth service/devices and regulation flexibility for virtual consulting will continue after the pandemic is over.

To help integrate telehealth into clinical practice and improve patient care, health policy at the systemic level should accelerate the uptake of telehealth. On the industry level, hospitals should identify adoption strategies for different types of telehealth services and evaluate telehealth products for health care delivery. On the physician level, health providers should offer the same level of care and follow the same treatment guidelines for telehealth services as with in-person visits and ensure that their practices are compliant with applicable regulations.

## Development of Telehealth

Telehealth has become a rapidly growing sector of health care delivery systems. Previous studies show evidence that telehealth tools and services increase the overall effectiveness of physicians in (1) counseling patients with chronic conditions, (2) psychotherapy support for behavioral interventions, and (3) remote monitoring of patients [2].

The shortage of health providers and increasing consumer demand (from the aging population and people diagnosed with chronic diseases) were key factors in expanding the scope and scale of telehealth services [3]. The 2019 annual report of the Association of American Medical Colleges projected a shortfall of 40,000 to 122,000 physicians in the United States over the next decade, with a shortage of 29,000 to 42,900 doctors in 2020 [4]. To use telehealth as a new strategy to stretch the physician supply, the Interstate Medical Licensure Compact standardized licensing requirements that allow physicians to practice in multiple states and provide remote digitalized services [5]. To further remove regulatory and reimbursement barriers to telehealth services, telehealth parity laws require commercial health insurers to provide equal coverage for telehealth and in-person services in 38 states and the District of Columbia [6]. By January 2017, all state Medicaid programs reimburse teleradiology, 49 cover tele–mental health services, and 36 states cover various remote telehealth services [2].

The COVID-19 pandemic accelerated the adoption of telehealth tools and services. Amid the pandemic, some hospitals are seeing 500 to 600 patients per day via video or telephone visits [7]. To enable providers to use telehealth services, Medicare implemented temporary payment flexibility to allow more beneficiaries to benefit from virtual care services and more providers to be eligible to bill for telehealth services at the same payment rate as they would receive for in-person services [8]. The Centers for Medicare and Medicaid Services (CMS) added 135 allowable services, including emergency department visits; initial nursing facility and discharge visits; home visits; and physical, occupational, and speech therapy services [9]. With these initiatives, US provider systems are rapidly deploying digitalized services for two main goals:

1. Forward triage to screen patients with COVID-19 symptoms before arrival to a health care facility so as to reduce exposure to the virus [10]
2. Continue patient care and provide virtual consultation to nonvirus patients, especially those with chronic diseases

## Telehealth Success Model

**Figure 1:**
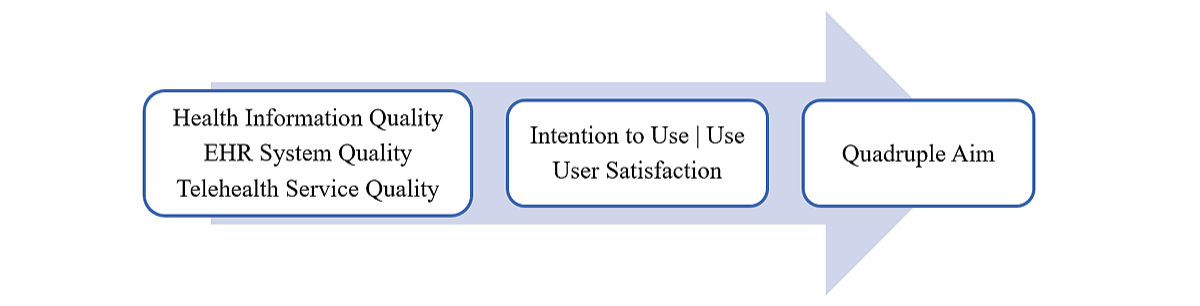
Telehealth success model. EHR: electronic health record.

This paper applies a conceptual model (Figure 1) based on Delone and McLean’s [11] model of information systems success to assess the impact of hospital medical record interoperability on telehealth service outcomes. As framed by Delone and McLean [11], a sustainable information system depends on positive results from the quality of information, service, and systems, as well as interrelated measures of user satisfaction, use, and net benefits.

## Health Information Quality

**Figure 2:**
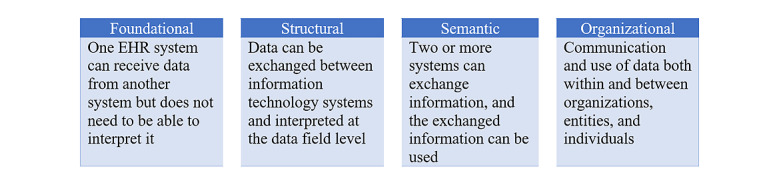
Hospital interoperability levels. EHR: electronic health record.

Device interoperability and data integration are key aspects of telehealth delivery. Hospital interoperability (Figure 2) covers three types of information exchange: (1) sending, receiving, and incorporating health records that support electronic referral loops; (2) electronic access for both physicians and patients to their health information; and (3) public health surveillance that collects and integrates health-related data to assist planning, implementing, and evaluating public health practice [12].

For value-based care to achieve and protect patient safety, the EHR system needs to deliver accurate and clinically appropriate data across care settings to both physicians and patients. Studies suggest that hospital sharing of diagnostic data with providers within their system is associated with lower patient mortality, and the hospital interoperability level is associated with improved process quality related to conditions of acute myocardial infarction, heart failure, and pneumonia at acute care hospitals [13-15].

However, establishing a telehealth platform in a short period of time amid pandemic conditions could put health information—patient names, address, dates, diagnoses, and more—at higher risks to safety and security [16]. The introduction of increasingly complicated technology into already complex work environments may trigger various unintended interactions that undermine or outweigh the potential benefits of the new technology [17]. Moreover, with an exponential growth in clinical data, it becomes critical to code symptoms (eg, allergies) and medications correctly to ensure patient safety and care quality [18].

## EHR System Quality: Maximizing the Benefits of Telehealth

Integrating telehealth programs into a hospital’s existing EHR system infrastructure helps maximize the benefits of telemedicine, as providers and staff already have experience working with the baseline system [19]. Over 95% of US hospitals reported using a certified EHR platform [12]. However, many hospitals run separate systems for doctors, labs, radiologists, and remote monitoring devices; the technical and data incompatibilities between different vendors make data sharing more vulnerable to cybersecurity threats [20]. Suboptimally integrated systems also added clerical burdens on physicians that can help lead to professional burnout. For every hour of clinical work, physicians spent 2 hours on EHR-related tasks, threatening the capacity and performance of the health system [21].

In 2020, the US Office of the National Coordinator for Health Information Technology (ONC) established requirements for a secure standards-based application programming interface (API) to support each individual patient’s access and control of their electronic health information [22,23]. The increasing data volumes, new data types, and various data sources collected from telehealth services can make it difficult and labor-intensive to match or identify the correct patient between systems [24]. Thus, a wide-scale adoption of common standards would drive data sharing and make integration more consistent and efficient, thereby providing clinically useful information and mitigating physician burnout [25].

## Telehealth Service Quality

Advanced interoperability—especially at semantic and organizational levels—can enable telehealth to expand access, exchange information, and provide user-centered services to both physicians and patients [26]. Current telehealth services use devices such as wearable monitors, smartphones, mobile apps, video, email, and web portals to deliver three types of care: (1) remote monitoring of patients and collecting vital signs and health data for care plan management, (2) counseling and interacting with patients at home, and (3) triaging patients to screen them to reduce exposures to viruses and thereby free up hospital resources during emergencies [27].

## Telehealth Use in Clinical Practice

### Rural Health Services

Approximately 80% of rural areas in the United States are classified as medically underserved and in health professional shortage areas [28,29]. These regions are lacking the physicians, registered nurses, and behavioral health providers (including psychiatrists, psychologists, and therapists) [30]. The patient-to-primary care physician ratio in rural areas is only 39.8 physicians per 100,000 people, compared to 53.3 in urban areas [31]. The shortage disproportionately impacts rural residents who tend to be older, have lower socioeconomic status, are more reliant on public insurance, and have worse health outcomes [32,33].

For rural residents, telehealth care increased access to experienced providers and high-quality care [34,35], improved continuity of care and health outcomes [36], and reduced health disparities [37]. Studies have shown that greater adoption of telehealth was associated with facilities in rural locations [38]. Between 2010 and 2017, telehealth visits have increased among rural Medicare beneficiaries, with a 425% increase for tele–mental health services [36].

### Tele–Mental Health Services

There are two primary uses for tele–mental health: provider consultations with mental health specialists in primary care and emergency department settings, and the direct provision of mental health services including home-based services [37,38].

For people experiencing serious mental illness, telehealth has the potential to improve quality of life and general mental health, reduce depressive symptoms, build more confidence in managing depression, and increase satisfaction with mental health and coping skills compared to treatment offered in-person only [36,39]. For people experiencing substance use disorders (SUDs), treatments delivered through telehealth have resulted in reductions in alcohol consumption, increased tobacco cessation, and increased engagement and retention in opioid use disorder treatment [36]. Between 2016 and 2019, SUD treatment offered through telehealth increased from 13.5% to 17.4% [40].

### Veterans’ Health Services

As one of the early adopters of telehealth, the Veterans Health Administration (VHA) is currently the largest telehealth provider in the United States [41]. In 2018, VHA conducted over a million telehealth visits [42]. A total 10% of the visits used VA Video Connect (VVC), a secure video teleconferencing platform that allows providers to treat veterans on their mobile devices or personal computers at a location of the veteran’s choice [43,44]. The 2018 “Maintaining Internal Systems and Strengthening Integrated Outside Networks” (“MISSION”) Act included mandates for VHA to establish an “Anywhere to Anywhere” telehealth network, where VHA providers in outpatient mental health and primary care service lines nationwide will be both capable and experienced with providing telehealth (VVC) to non-VA locations [43]. The demands for VA telehealth services also increased during the pandemic [45]. Tele–mental health sessions via VVC increased 42% at one VA medical center in South Carolina, from 1429 appointments in January 2020 to 2034 in March 2020 [43,45].

### Patient-Centered Care

A telehealth program paired with the right EHR system can serve as a care collaboration platform and help optimize team-based care delivery [13,46]. It connects off-site specialists in the fields of cardiology, psychiatry and behavioral health, oncology, and infectious disease with patients at home or intensive care units (ICUs) [2]. Physicians can easily access and send health records from one interface to another (mobile, computer, or tablet) remotely using a protected account to diagnose and assess symptoms as an in-person consultation [47].

Telehealth interventions, particularly remote monitoring and SMS text messaging, were associated improvements in obstetric outcomes, perinatal smoking cessation, breastfeeding, and schedule optimization for high-risk obstetrics [48]. For at-risk patients with chronic disease, remote monitoring devices continuously capture physiological data such as heart rate, blood glucose, oxygen saturation level, body temperature, blood pressure, and weight over time [2,49]. Evidence also suggests telehealth services allowed physicians to better communicate with patients on treatment plans that are appropriate for their culture, race, gender, sexual orientation, and lived experience [35,48,50].

### Population Health Management

An EHR system with organizational interoperability allows institutions to aggregate community-level data from disparate sources to track influenza/disease trends for population health [51,52]. For example, CMS requires hospitals to electronically report public health data such as syndromic surveillance data, electronic case reporting, reportable laboratory results, and more. Sharing critical data among health care systems, especially during a pandemic, assists public health authorities to predict clusters of outbreaks and make timely and efficient guidance for quarantine and better containment [53,54].

## Postpandemic Health Care Needs and Challenges

### Regulation Uncertainties

The COVID-19 pandemic in the United States is affecting different areas at different times and levels: cases spike in some states while others face the threats of both COVID-19 resurgence and seasonal flu [55]. Many hospitals are providing a combination of traditional in-person visits and telehealth services that allow remote virtual consultations to patients [34].

Early in March 2020, CMS modified policies to lift originating site restrictions and expand the type of visits allowed virtually [56]. However, after the pandemic, hospitals may shift telehealth services from urgent care and COVID-19 screenings and treatment to regular care visits [57]; it is unclear how current more flexible regulation and payment arrangements will change [56,58]. Will there be permanent polices to reimburse virtual care and remote monitoring devices? Will telehealth reimbursement rates be set at the same level with in-person visits? Will there be financial incentives to provide reliable broadband access to rural or small hospitals [59]?

With hospitals and the health care system faced with uncertainties about the duration of this pandemic and the structure of future telehealth benefits, the development of clear regulatory requirements and timetables could help reduce administrative and technological constraints associated with virtual health care delivery and encourage further investment in health care information technology (IT) infrastructure [60,61].

### Hospital Financing Challenges

The COVID-19 pandemic has created substantial financial difficulties for both hospitals and the health system [62,63]. As a result of cancelled elective surgeries and nonessential medical procedures, which often generate more revenues than ICU and emergency care, US hospitals continue to experience substantial losses in revenue [64]. Expenses also have increased sharply from purchasing needed for personal protective equipment, COVID-19–associated hospitalizations, and providing additional support to frontline health workers [62]. The American Hospital Association estimates a total 4-month financial impact of US $202.6 billion in losses for US hospitals and health systems, or an average of US $50.7 billion per month [62]. These financial loss and additional system maintenance/implementation costs for telehealth and EHR systems will require decision makers to establish more effective strategies to use hospital resources and workforce [64,65].

## Disparities in Telehealth Access

Although most hospitals in the United States have adopted interoperable EHR systems, there is little evidence about whether small, rural, and safety net hospitals are keeping up [66]. Compared to more technologically advanced hospitals, smaller and rural hospitals have limited broadband access [59], less interoperability and health care IT management experience [67], and staff with less technological familiarity [68,69]. Because of the uneven adoption of telemedicine services, some small clinics and postacute care facilities are unable to receive or share patient data [70,71].

Substantial disparities in access to telehealth services also remain [72]. Evidence suggests geographical disparities, profit-based discrimination, technology deployment cost, and socioeconomic factors played key roles in the telehealth use gap [59,72,73]. Moreover, people 65 years and older, with disabilities, experiencing poverty, and who are non-White are less likely to use telehealth services because of lower smartphone or computer ownership, limited (home) broadband internet access, and low digital literacy [36]. A recent study of 134,225 completed primary care visits also reported that early adopters of online scheduling were more likely to be young, White, and commercially insured [74].

## Regulatory Process for EHR Market

To ensure that EHRs cooperate effectively in an interoperable structure, substantial governmental regulation has been put into place. In the United States, CMS and the ONC regulate EHR privacy, security, and standards for hospitals or health providers and health IT developers [75,76]. In July 2021, the Interoperability and Patient Access final rule began to require CMS-regulated payers to remove industry siloes and support Patient Access API, Provider Directory API, and Payer-to-Payer Data Exchange to achieve greater semantic interoperability within the health care system while complying with existing Health Insurance Portability and Accountability Act (HIPAA) requirements [75,77]. On the technical level, CMS adopted Health Level 7 Fast Healthcare Interoperability Resources Release 4.0.1 to standardize implementing privacy and security features for provider organizations [78].

In the European Union, to facilitate cross-border EHR interoperability, the General Data Protection Regulation established explicit rules to process and protect patient health data [79]. On the technical level, the eHealth Digital Service Infrastructure has enabled provider organizations to exchange patient summaries and e-prescriptions [78]. In the Asia-Pacific region, Singapore, Japan, and Australia have instituted regulations on software qualification, software as a medical device, and presubmission consultation by regulatory authorities to facilitate EHR interoperability [80-82]. In Singapore, for example, the National Electronic Health Record system sets technical standards (including architecture, security, and operations) for the digital health market and monitors user functionality and risk to protect data security [83].

## Discussion

Studies suggest that telehealth programs paired with the right EHR system enhance care access, increase patient satisfaction, and reduce medical spending [84,85], and by improving clinician experience, the integrated system can contribute to achieving the quadruple aim.

To help integrate telehealth into clinical practice and improve patient care, on a systemic level, health policies should accelerate the uptake of telehealth, including tele–mental health, to improve care quality, cost-effectiveness, and value of care [86]. Federal and state governments can use disruptive reimbursement and funding strategies on training primary and mental health care providers, workforce, licensure, and cultural sensitivity for long-term telehealth practice [34,87]. Regulators also need to assess and set standards for malpractice liability and protect patient safety and confidentiality that may result from telehealth deployment [88].

On an industry/organization level, hospitals need to identify strategies to adopt and integrate different types of telehealth services, and evaluate telehealth products for health care delivery [66,70]. Future studies are needed to provide evidence on telehealth practice guidelines and service models.

On the physician level, clinicians who provide telehealth should offer the same level of care and follow the same treatment guidelines they would follow for in-person visits [89]. Moreover, physicians should closely follow HIPAA rules, state laws, and medical board definitions to ensure their practices are compliant with applicable regulations while implementing telehealth [57].

## Abbreviations

API: application programming interface
CMS: Centers for Medicare and Medicaid Services
EHR: electronic health record
HIPAA: Health Insurance Portability and Accountability Act
ICU: intensive care unit
IT: information technology
MISSION: Maintaining Internal Systems and Strengthening Integrated Outside Networks
ONC: Office of the National Coordinator for Health Information Technology
SUD: substance use disorder
VHA: Veterans Health Administration
VVC: VA Video Connect
